# Craniofacial photography for detection of positional obstructive sleep apnoea

**DOI:** 10.1007/s41105-025-00602-y

**Published:** 2025-07-31

**Authors:** Kate Sutherland, John Wheatley, Kristina Kairaitis, Brendon J. Yee, Gary Cohen, Kerri Melehan, Stephen Lambert, Philip de Chazal, Peter A. Cistulli, The Sydney Sleep Biobank Investigators, Kate Sutherland, John Wheatley, Kristina Kairaitis, Gary Cohen, Kerri Melehan, Stephen Lambert, Peter Cistulli, Philip de Chazal, Nina Sarkissian, Yu Sun Bin, Chin Moi Chow, Andrew Chan, Aimee Lowth, Jacob Graham, William Wood, Callum Bennett, Mohammad Ahmadi, Rita Ginn, Tracey Burns, Brendon Yee, Amanda Piper, Keith Wong, Margaret Chan, David Wang, Gislaine Gauthier

**Affiliations:** 1 Sleep Research Group, Charles Perkins Centre, University of Sydney, Camperdown, NSW Australia; 2 Sydney Medical School, Faculty of Medicine and Health, The University of Sydney, Sydney, NSW Australia; 3 Ludwig Engel Centre for Respiratory Research, The Westmead Institute for Medical Research Westmead, Westmead, NSW Australia; 4 Department of Respiratory and Sleep Medicine Westmead Hospital Westmead, University of Sydney Westmead, Westmead, NSW Australia; 5 Department of Respiratory and Sleep Medicine, Royal Prince Alfred Hospital, Camperdown, NSW Australia; 6 Woolcock Institute of Medical Research, Sydney, Australia; 7 School of Biomedical Engineering, Faculty of Engineering, The University of Sydney, Darlington, NSW Australia; 8 Sleep Investigation Unit, Department of Respiratory Medicine, Royal North Shore Hospital, Level 8A, St Leonards, NSW 2065 Australia

**Keywords:** Obstructive sleep apnoea, Supine position, Craniofacial phenotype

## Abstract

Supine-dependent OSA is a well-recognised OSA phenotype and may relate to craniofacial structure. Our aim was to assess whether craniofacial photos are able to identify positional OSA, specifically supine-isolated OSA, in comparison to non-positional OSA. Frontal and profile craniofacial photographs of participants were acquired according to a standardised protocol. Photographs were analysed and compared between non-positional and supine-isolated OSA groups. A total of 156 OSA patients were included (54.5% supine-isolated OSA, 45.5% with non-positional OSA). The supine-isolated group had a longer upper face height and greater upper-to-lower face height ratio, smaller face width, reduced face width-to-height ratio, and smaller mandibular width, length, and size of the mandibular base. Differences in facial measurements were no longer significant after adjustment for body size and OSA severity. Our study demonstrates that supine-isolated OSA can be identified using facial photography. Larger studies in groups matched for BMI and OSA severity are needed to confirm whether this technique may also capture other features related to supine-isolated OSA (i.e., differences in underlying skeletal structure).

## Introductions

Obstructive Sleep Apnoea (OSA) is estimated to affect nearly 1 billion people globally and is associated with a range of comorbidity including daytime symptoms, reduced quality of life, neurocognitive impairment, and cardiometabolic diseases. OSA is characterised by repetitive collapse of the pharyngeal airway during sleep; however, the causes, characteristics, and consequences of this vary between individuals. Understanding OSA clinical phenotypes has implications for screening strategies and personalised management of this disorder to improve outcomes [1].

Craniofacial structure is a risk factor for OSA and has implications for the success of different treatment modalities [2–4]. Traditionally capturing detailed craniofacial information to assist in OSA screening and treatment decisions in the clinical setting has been impractical via radiographic and magnetic resonance imaging modalities due to issues of access and cost. Facial photography is a simplified way to capture craniofacial phenotypic information which has been shown to be related to OSA risk across ethnicity and gender [5]. Craniofacial structures captured by this method have been shown to predict OSA treatment outcomes, including weight loss, oral appliance therapy, and CPAP adherence and mask fitting [2–4]. Mechanistic studies have shown that photos capture useful information about upper airway structures, and although it represents a composite of skeletal and soft tissue, some measures are more reflective of individual components [6]. Further exploration of OSA phenotyping using facial photography is needed to understand its utility as a tool for this purpose.

One long recognised phenotype of OSA is supine position dependency where obstructed breathing events are either isolated to or predominantly occur in the supine body position. Supine-dependent OSA is found in at least half of the OSA cases presenting to sleep clinics, and half of those can be classified as OSA isolated to the supine position (supine-isolated OSA) [7]. Clinical characteristics of supine-dependent OSA include less obesity, younger age, and lower overall AHI relative to those with non-positional OSA [7]. The pathogenesis of supine-dependent OSA is not well understood, but there is suggestion that craniofacial structure may be one predisposing factor. Whether facial phenotyping using the photographic method conveys information about this phenotype of OSA is unknown. Our aim was to assess whether craniofacial photos are able to identify positional OSA, specifically supine-isolated OSA, in comparison to non-positional OSA.

## Methods

**Fig. 1:**
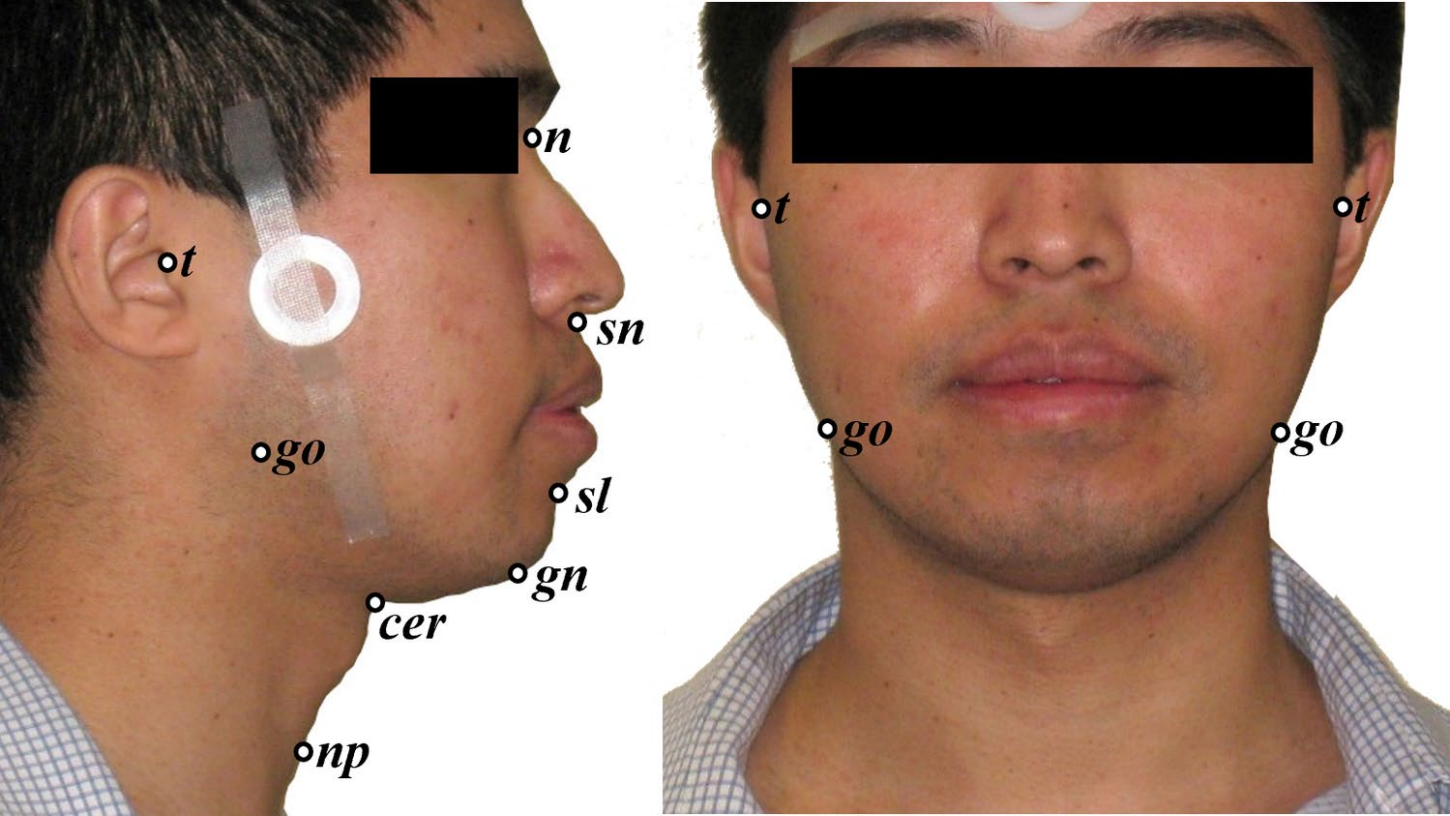
Craniofacial landmarks used to calculate measurements from front and profile facial photographs. The landmarks used in this analysis are nasion (*n*), subnasion (*sn*), menton (*me*), tragion (*t*), gonion (*go*), sublabiale (*sl*), neck plane point (*np*), and cervical point (*cer*). The circular nylon calibration marker which allows quantitative measurements to be obtained can be seen on the side of the face. Measurement definitions: upper face height = *n-sn*; lower face height = *sn-me*; face width = *t-t*; cranial base triangle area = *t-n-t*; mandibular width = *go-go*; mandibular length = *gn-go*; mandibular angle = *t-go-gn*; mandibular base angle = *go-me-go*; mandibular base angle = *go-me-go*; maxilla depth angle = *t-n-sn*; mandible depth angle = *t-n-sl*; maxilla–mandible relationship angle; *sn-n-sl*; cervicomental angle = *np-cer-me*

Data were obtained from the Sydney Sleep Biobank (SSB) [8]. The SSB is a prospective data collection of participants attending for sleep studies at one of three tertiary hospital sleep laboratories in Sydney, Australia (Royal North Shore Hospital, Royal Prince Alfred Hospital, and Westmead Hospital). Adults (> 18 years) attending for polysomnography for suspected sleep-disordered breathing are eligible for participation in the SSB. SSB data collection includes demographic, anthropometric, polysomnography data, standardised craniofacial photographs, questionnaires, and biological samples. Ethics approval has been granted by the Northern Sydney Local Health District (NSLHD) Human Research Ethics Committee (HREC), protocol number HREC/17/HAWKE/340. All participants gave written informed consent to contribute their data to the SSB. Frontal and profile craniofacial photographs of participants were acquired according to previously described protocols [2, 3, 5, 10] and depicted in Fig. 1. This facial photography protocol has been developed to acquire images standardised manner in a way that is simple to perform in any setting. Briefly, to ensure standardisation, the subject is instructed to assume a natural head position by ‘imagine looking into your own eyes in a mirror’ and told to relax with a neutral facial expression with lips and teeth lightly touching. The photographer takes the image from directly in front of the face and a solid circular calibration marker allows for quantitative measurements to derive without the need for the image to be taken from a set distance. At the time of analysis, there were 421 SSB participants with craniofacial photos available for analysis. Those with OSA (defined as AHI ≥ 5 events/hour), sufficient total sleep time (> 3 h), and at least 15 min sleep in both supine and non-supine body positions during the study were considered for this analysis. As it has been shown that supine-dependent OSA can vary night to night when broadly defined, we chose to select a supine-isolated definition (AHI_Supine_: AHI_Non-Supine_ ratio ≥ 4 plus AHI_Non-Supine_ < 10 events/hour) as there is evidence this is a more stable phenotype [9]. Therefore, we compared craniofacial phenotype in a supine-isolated OSA group to a comparator group with non-positional OSA (AHI_Supine_: AHI_Non-Supine_ < 2). These groups were compared using independent t tests (or non-parametric equivalent as appropriate) and additionally using an ANVOCA model to adjust for covariates. We chose to assess the difference in raw facial measurements between positional OSA groups and also with adjustment for BMI, neck circumference, and AHI. Positional OSA groups are known to be different on these characteristics and our previous work also demonstrates that facial measurements pick up differences in body size and OSA severity [2, 3, 5, 10]. Therefore, we wished to explore whether facial measurements may relate to positional OSA through these group differences or show a relationship independent of these factors. Statistical analysis was performed using IBM SPSS Statistics Version 27 (Armonk, NY:IBM Corp).

## Results

A total of 156 OSA patients were eligible for inclusion in this analysis. There were *N* = 85 (54.5%) OSA patients identified with supine-isolated OSA and *N* = 71 with non-positional OSA. The characteristics of the supine-isolated and non-positional groups are shown in Table 1. The supine-isolated group had lower body mass index (BMI) and neck and waist circumferences. Craniofacial measurements in the supine-isolated and non-positional OSA groups are shown (Table 1). For craniofacial measurements in the face region, the supine-isolated group had a longer upper face height and hence greater upper-to-lower face height ratio. Face width was on average smaller than the non-positional OSA group with a reduced face width-to-height ratio. For craniofacial measurements reflecting the mandible region, the supine-isolated group again had smaller mandibular width, length, and size of the mandibular base (Table 3). There were no differences between supine-isolated and non-positional OSA groups in measurements reflecting the relative position of the maxilla and mandible. The cervicomental angle of the neck was reduced in the supine-isolated group, indicative of less submental soft tissue than the non-positional group. In analyses adjusted for covariates including the differences in body size measures between groups (BMI and neck circumference) and total AHI, none of the craniofacial measurement differences remained statistically significant. The findings remained the same when only including covariates for body size in the analysis.

**Table 1:** Comparison of OSA patients with supine-isolated and non-positional phenotypes

	Supine-isolated OSA	Non-positional OSA	P value	P value *Adjusted^*
*N*	85 [54.5]	71 [45.5]		
Demographics				
Age (years)	56.0 (17.0)	56.0 (22.0)	0.709	
Gender (M/F/T)	62/22/1	50/21	0.588	
Ethnicity			0.694	
White	57 [67.1]	36 [50.7]		
Indigenous Australian	1 [1.2]	3 [4.2]		
Pacific Islander	3 [3.5]	4 [5.6]		
Asian	11 [12.9]	14 [19.7]		
Middle Eastern	3 [3.5]	3 [4.2]		
Central/South American	4 [4.7]	2 [2.8]		
Anthropometry				
Height (cm)	174.1 (13.8)	172.5 (10.0)	0.065	
Weight (kg)	87.2 (18.6)	106.0 (48.0)	< 0.001*	
BMI (kg/m^2^)	29.3 (6.9)	36.2 (15.1)	< 0.001*	
Neck circumference (cm)	40.4 ± 3.6	44.2 ± 5.6	< 0.001*	
Waist circumference (cm)	102.8 (12.9)	118.4 (31.5)	< 0.001*	
Hip Circumference (cm)	107.0 (12.2)	118.9 (26.5)	< 0.001*	
Modified Mallampati (N[%])				
Grade 1	9 [10.6]	5 [7.0]	0.582	
Grade 2	4 [4.7]	9 [12.7]		
Grade 3	19 [22.4]	22 [31.0]		
Grade 4	53 [62.4]	35 [49.3]		
Polysomnography				
Sleep efficiency (%)	79.9 (17.3)	77.9 (19.6)	0.783	
Total sleep time (min)	365.6 ± 70.8	377.3 ± 70.8	0.405	
Supine sleep time (min)	155.5 (136.4)	176.5 (151.0)	0.165	
AHI (events/hour)	14.9 (16.5)	56.5 (53.4)	< 0.001*	
AHI_NS_ (events/hour)	2.6 (3.8)	48.4 (52.5)	< 0.001*	
AHI_S_ (events/hour)	39.3 (35.5)	62.8 (67.2)	0.005*	
AHI_S_:AHI_NS_ ratio	11.1 (15.4)	1.1 (0.5)	< 0.001*	
Facial measurements				
Upper face height (cm)	5.1 (0.6)	4.9 (0.6)	0.004*	0.341
Lower face height (cm)	6.8 (0.9)	6.8 (0.9)	0.176	0.591
Upper/lower face height (ratio)	0.8 (0.1)	0.7 (0.1)	0.002*	0.682
Face width (cm)	12.2 ± 0.8	12.9 ± 1.0	< 0.001*	0.674
Face width/height (ratio)	1.0 ± 0.1	1.1 ± 0.1	0.001*	0.389
Cranial base triangle area (cm^2^)	64.1 ± 6.1	66.0 ± 8.7	0.124	0.903
Mandibular width (cm)	9.6 (1.2)	10.8 (1.9)	< 0.001*	0.929
Mandibular length (cm)	9.2 ± 0.8	9.5 ± 1.1	0.033*	0.254
Mandibular length/width (ratio)	0.9 (0.1)	0.9 (0.1)	0.002*	0.368
Mandibular angle (°)	122.6 (10.3)	120.8 (10.0)	0.248	0.981
Mandibular base angle (°)	69.4 ± 7.3	72.8 ± 10.8	0.006*	0.368
Mandibular base area (cm^2^)	33.0 (9.5)	37.9 (9.4)	0.003*	0.270
Maxilla depth angle (°)	81.5 (6.9)	80.6 (7.7)	0.841	0.507
Mandible depth angle (°)	73.7 (6.0)	74.3 (8.0)	0.544	0.511
Maxilla–mandible relationship angle (°)	7.5 (2.9)	7.2 (3.6)	0.152	0.974
Cervicomental angle (°)	152.8 (20.3)	171.3 (29.2)	< 0.001*	0.249

*AHI* apnoea–hypopnoea index, *F* female, *M* male, *T* transgender male, *NS* non-supine, *S* supine

OSA patients identified to be supine-isolated (AHI_S_: AHI_NS_ ratio ≥ 4 and AHI_NS_ < 10 events/hour) and non-positional (AHI_S_: AHI_NS_ ratio < 2) were compared on demographic, anthropometric, and polysomnographic variables. Data are presented as mean ± standard deviation for normally distributed continuous variables, Median (interquartile range) for non-parametric continuous variables, and N [%] for categorical variables. Supine-isolated and non-positional groups were compared using independent t test (Mann–Whitney U tests for non-parametric variables) or Chi-square test for categorical variables. Craniofacial measurements presented in four regions, for supine-isolated and non-positional OSA patients. Data are presented as mean ± standard deviation for normally distributed variables, Median (interquartile range) for non-parametric variables. The P value is obtained from independent t tests or Mann–Whitney U tests for non-parametric variables as appropriate. The adjusted P value is from ANCOVA models with body mass index, neck circumference, and apnoea–hypopnoea index as covariates in comparison of craniofacial measurements between the supine-isolated and non-positional groups. *P < 0.05 was considered significant. ^*P* value from ANCOVA model adjusted for body mass index, neck circumference, and apnoea–hypopnoea index

## Discussion

Using assessments of craniofacial phenotypic information, obtained using quantitative facial photography, we demonstrated differences in craniofacial phenotype between supine-isolated and non-positional OSA. In particular, the supine-isolated cohort had smaller craniofacial dimensions. Given that these observed craniofacial difference do not remain after adjustment for obesity, it suggests that they are primarily explained by different obesity levels between these OSA clinical phenotypes. Overall, it appears that any craniofacial phenotypic differences between these groups seem to be due to differences in body size. It may not be possible to detect craniofacial skeletal differences between these OSA clinical phenotypes using this method due the inherent clinical differences between these groups which also influence facial measures. Therefore, conclusions about craniofacial skeletal differences and an anatomical basis of supine-dependent OSA cannot be made from this study. However, facial analysis is picking up differences in these clinical characteristics related to the supine-isolated OSA phenotype.

There were no differences in demographics (age, gender, and ethnicity) in our cohorts. Our findings of lower BMI with smaller body circumferences in supine-isolated OSA are well reported. It is yet unclear whether these anthropometric differences could be explained by supine-isolated OSA being on the pathway from snoring to OSA in all positions which may be progressed by increasing fat deposition around the chest and upper airway, or whether supine-isolated OSA is a separate OSA phenotype altogether. The supine-isolated group also had much less severe OSA based on total AHI (on average only mild OSA) compared to the non-positional group. BMI and OSA severity are also related to facial characteristics [2] and hence are confounders in comparing craniofacial characteristics between OSA clinical phenotypes in this sample. To more completely understand if positional OSA is identifiable in facial phenotype beyond these factors, a matched sample on body size/fat distribution and OSA severity would be desirable. Craniofacial measurements also differ by ancestry/race and OSA risk also varies with ancestral background [10]. Although there was no statistical difference in the ethnicity profile between the OSA groups in our cohort, the sample is majority White ethnicity (~ 60%). Although other ethnicities were recruited, in this sample, we did not have sufficient numbers to stratify analysis by ethnicity. Therefore, this is a limitation to the current work. It could be that facial measurements relate more directly to positional OSA phenotypes in specific ethnic groups, such as Asian populations where craniofacial factors have been previously shown to be more associated with OSA risk than soft-tissue factors [5]. Therefore, there could be differences in these craniofacial variables that associate with positional OSA by ethnicity which would need to be explored in larger samples; however, matching on ethnicity and body size was not possible in the sample available and this is a limitation of this analysis.

Facial analysis is a non-invasive and increasingly feasible tool to apply to clinical settings and large-scale research given advances in technologies for image capture and automated analysis. Facial phenotyping has been shown to predict OSA risk and relate to OSA treatment outcomes [2–5]. In recent years, there has been growing interest in understanding OSA phenotypes for potential to improve outcomes via individualised management [1]. Craniofacial structure and position dependency are phenotypes of OSA that have implications for diagnosis and treatment. In this study, we explored whether facial analysis could have application in identifying OSA phenotypes, in this case position dependency, as well as potential to explain differences in how these phenotypes generate, i.e., underlying craniofacial structure. Although this work is preliminary, it highlights further potential for OSA phenotyping and the richness of information that could be acquired towards individualised management using facial analysis technology.

## Conclusion

Facial photography allows for quantitative craniofacial information to be obtained in large samples and is a simple tool for OSA phenotyping and therefore has potential for clinical use in individualised diagnosis and treatment of OSA. Facial analysis shows differences between body position phenotypes of OSA. Therefore supine-isolated OSA could be identified using facial photography. Larger studies of facial photography in groups matched for BMI and OSA severity would be needed to confirm whether this technique may also capture other features related to supine-isolated OSA (i.e., differences in underlying skeletal structure). Ethnicity may also be an important factor in association between facial and OSA phenotypes and requires further investigation.
